# Wearable Device Oriented Flexible and Stretchable Energy Harvester Based on Embedded Liquid-Metal Electrodes and FEP Electret Film

**DOI:** 10.3390/s20020458

**Published:** 2020-01-14

**Authors:** Jianbing Xie, Yiwei Wang, Rong Dong, Kai Tao

**Affiliations:** 1 Key Laboratory of Micro/Nano Systems for Aerospace, Ministry of Education, Northwestern Polytechnical University, Xi’an 710072, China; wangyw@mail.nwpu.edu.cn; 2 School of Mechatronic Engineering, Xi’an Technological University, Xi’an 710021, China; dongrong1981@163.com

**Keywords:** flexible and stretchable electronics, energy harvester, liquid-metal, wearable device, electret

## Abstract

In this paper, a flexible and stretchable energy harvester based on liquid-metal and fluorinated ethylene propylene (FEP) electret films is proposed and implemented for the application of wearable devices. A gallium liquid-metal alloy with a melting point of 25.0 °C is used to form the stretchable electrode; therefore, the inducted energy harvester will have excellent flexibility and stretchability. The solid-state electrode is wrapped in a dragon-skin silicone rubber shell and then bonded with FEP electret film and conductive film to form a flexible and stretchable energy harvester. Then, the open-circuit voltage of the designed energy harvester is tested and analyzed. Finally, the fabricated energy harvester is mounted on the elbow of a human body to harvest the energy produced by the bending of the elbow. The experimental results show that the flexible and stretchable energy harvester can adapt well to elbow bending and convert elbow motion into electric energy to light the LED in a wearable watch.

## 1. Introduction

With the development of wearable devices, flexible and stretchable power supplies have become key technologies [1,2]. In recent years, several types of stretchable strain sensors have been proposed using nanomaterials coupled with flexible and stretchable elastomers. To improve stretchability, conductive particles such as nanoparticles (NPs) [3,4,5,6], carbon nanotubes (CNTs) [7,8,9,10,11,12], silver nanowires (AgNWs) [13,14,15], and graphene [16,17] are typically coated on or incorporated into a soft elastomer such as polydimethylsiloxane(PDMS), Ecoflex [14,18], silicone elastomer [19,20], rubber [21,22], or dragon-skin elastomer [23]. In addition, there are also some energy harvesters that are made of an origami structure to collect marine energy [24]. Most of the previous studies used different materials to make energy harvester in different environments. They were affected by the environment, and the materials used were relatively different to obtain, and most importantly, their service life is not long enough. Compared with the previous energy harvester and sensors [24,25,26,27], we proposed to make flexible wearable energy harvesters with liquid metal alloy electrodes. We proposed to use liquid-metal alloy and fluorinated ethylene propylene (FEP) as electrode to design energy harvester and verify its feasibility. The FEP is bought on the internet, and the company’s name is GOOD FELLOW. Now there are many deficiencies, and we are trying to improve them. Compared with the previous research, our research is only applicable to collect the mechanical energy generated by human joint movement, only the stretching of the electrode, which cannot achieve the stretching of the whole device, but also needs to design a new structure to improve the whole stretching performance of the device. For example, the conductive electrode is designed in a form that can be stretched, and the two electrodes are bonded together.

Liquid metals are the most flexible and stretchable electrical conductors due to the fluidity of the liquid; they are both intrinsically deformable and highly conductive and have been shown to maintain metallic conductivity up to 700% strain [28]. In addition, liquid metals can be 3D printed, molded, or injected into sealed cavities; therefore, they are widely used in the field of flexible electronics, such as in soft electrodes [29,30], interconnects [31,32], microfluidics [33], sensors [11,15], and e-skin [12]. Therefore, the feasibility of using liquid metals as stretchable electrodes is verified in this paper.

**Figure 1:**
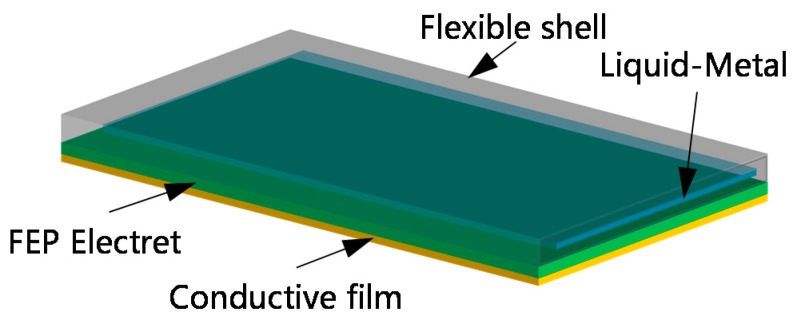
Structure of the flexible and stretchable energy harvester based on gallium liquid-metal alloy.

In this paper, we propose a flexible and stretchable energy harvester based on a gallium liquid-metal alloy, and this kind of alloy is composed of gallium and indium, and we bought them on the internet. The melting point of the alloy is different with different proportion of gallium and indium. The composition of the Ga-In alloy we used is 95Ga/5In, the melting point is 25 °C. In fact, the melting point of Ga-In alloy is between 25 and 30 °C. When the Ga is a solid state, it can not be stretched. Conductive film is a kind of film that the surface is full of Ag nano wires. The Ag nanowire film we used is purchased. Its substrate is PET, after the hardening treatment, the surface is pressed at high temperature to attach the nano silver wire to the substrate surface. In our design, we use 3D printing technology to print the mold, which the length is 102 mm, width is 52 mm, and thickness is 12 mm. We injected silicone rubber into the mold, took it out after the silicone rubber solidifies, got the silicone rubber mold with a cavity, inject the Ga-In alloy into the cavity, and then seal the alloy into the silicone rubber shell with silicone rubber. The version of the silicone rubber is Dragon Skin 10. The FEP film did not bond to the elastomer. They are independent of each other. The conductive film is a kind of film with Ag nano wires on its surface. We use double-sided adhesive to stick the conductive film and electret, but not the elastomer. The brand of double-sided adhesive is 3 M. The electrode in the solid state is wrapped in a dragon-skin silicone rubber shell to form a flexible and stretchable electrode. Then, it is bonded with fluorinated ethylene propylene (FEP) electret film and conductive film to form a flexible and stretchable energy harvester, as shown in Figure 1. The selected dragon-skin silicone rubber has a maximum strain approaching 900%, so it can provide enough deformation for the designed flexible electrode.

**Figure 2:**
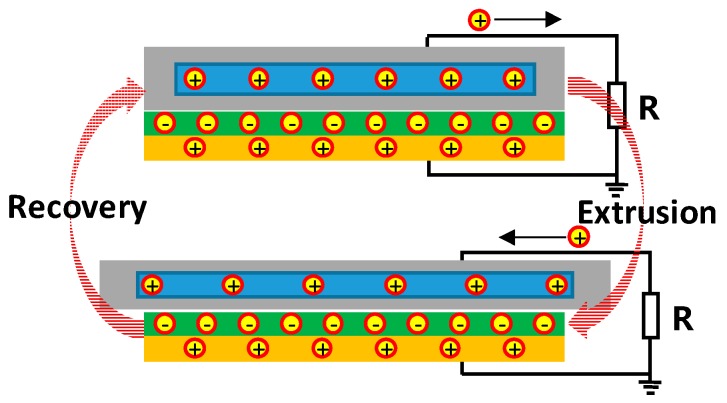
Energy conversion schematic of flexible and stretchable energy harvester based on gallium liquid-metal alloy.

When the stretchable energy harvester is squeezed, the elastomer will be deformed. The experiment principle of this design is that the charged electret induces the charge on the upper and lower electrodes to form a charged capacitance of the electrode plate. By charging the distance between the two plates, the device will supply power. According to the principle of electrostatic induction, a current will be generated in the load, as shown in Figure 2.

## 2. Design and Fabrication

In this paper, a gallium liquid-metal alloy was used as the stretchable electrode, which had a melting point of 25.0 °C. The transducer was composed of a low-temperature alloy wrapped in silicone rubber and a combination of electret and conductive film to form a variable capacitance. The electret itself was charged, so changing the distance between the two plates would change the capacitance and thus generate the directional movement of electrons, namely, current. The external applied force was provided by a shaking table and changed the acceleration of the shaking table to change the generating capacity of devices in different environments.

The calculation formula of voltage was deduced by charge conservation. Since the equipment was designed by charging and discharging of capacitance, the output voltage was calculated by capacitance value and charge quantity.

The charge $Q_{PET}$ in the electret would generate induced charges $Q_{Var}$ on the surface close to the electret in the liquid-metal alloy and $Q_{die}$ on the surface of conductive film. In addition, the generated surface triboelectric charges $Q_{TE}$ were stored on the surface of FEP.

(1) $$E_{PET}\varepsilon_{FEP}\varepsilon +E_{var}\varepsilon_{var}=-Q_{TE}/\left(\varepsilon_{0}S\right),$$

(2) $$S\varepsilon_{0}\varepsilon_{air}E_{Var}-Q_{Var}=0,$$

(3) $$S\varepsilon_{0}\varepsilon_{tape}E_{tape}-Q_{die}=0,$$

where $\varepsilon_{air}$, $\varepsilon_{PET},$ and $\varepsilon_{tape}$ are the relative permittivity of the air, FEP layer, and adhesive tape, respectively, and $\varepsilon_{0}$ is the permittivity of the vacuum. $E_{var}$, $E_{PET},$ and $E_{tape}$ are the electric fields in the air gap, the FEP layer, and in the adhesive tape, respectively. The charge conservation principle requires the following relation between $Q_{var}$, $Q_{die},$ and $Q_{TE}$.

(4) $$Q_{TE}+Q_{var}+Q_{die}=0.$$

Using Poisson’s equation for the three charge distributions, we find the following:

(5) $$V=-\int_{0}^{d_{tape}}E_{tape}dx-\int_{d_{tape}}^{d_{tape}+d_{PET}}E_{die}dx+\int_{d_{tape}+d_{PET}}^{d_{tape}+d_{PET}+d_{var}}E_{var}dx=-E_{tape}d_{tape}-E_{PET}d_{PET}+E_{var}d_{var}.$$

After putting Equations (1)–(4) into Equation (5), we obtained:

(6) $$V=\frac{Q_{TE}}{S\varepsilon_{0}}\left(\frac{d_{PET}}{\varepsilon_{PET}}+\frac{d_{tape}}{\varepsilon_{tape}}\right)+\frac{Q_{var}}{S\varepsilon_{0}}\left(\frac{d_{PET}}{\varepsilon_{PET}}+\frac{d_{tape}}{\varepsilon_{tape}}+\frac{d_{var}}{\varepsilon_{air}}\right)$$

Equation (6) can be further simplified by the following definitions:

(7) $$C_{EH}=\varepsilon_{0}S\frac{1}{\left(\frac{d_{PET}}{\varepsilon_{PET}}+\frac{d_{tape}}{\varepsilon_{tape}}+\frac{d_{var}}{\varepsilon_{air}}\right)},$$

(8) $$C_{die}=\varepsilon_{0}S\frac{1}{\frac{d_{PET}}{\varepsilon_{PET}}+\frac{d_{tape}}{\varepsilon_{tape}}},$$

(9) $$V_{TE}=\frac{Q_{TE}}{C_{die}},$$

where $V_{TE}$ is a constant voltage, $C_{die}$ is the total capacitance of the two dielectric layers with effective thicknesses of ddie=dPETεPET+dtapeεtape, and $C_{EH}$ is the total variable capacitance of the energy harvester.

Finally, this device was modeled by the following formula:

(10) $$V=-V_{TE}+\frac{Q_{var}}{C_{EH}}.$$

Under the periodic vibration of the shaking table in different gravity accelerations, the shaking table can change the frequency and amplitude of its vibration by setting. We used screws to fix the shaking table to the foam table below, and the function of the foam is to reduce vibration. The calculation formula of voltage was deduced by charge conservation. Since the equipment is designed by charging and discharging of capacitance, the output voltage was calculated by capacitance value and charge quantity.

The distance between the two electrodes ($d_{var}$) keeps changing between the maximum and minimum values, thus causing the capacitance to keep changing between the maximum and minimum values $C_{min}\leq C_{EH}\leq C_{max}$. Therefore, we obtained an important parameter of the energy harvester denoted as the “capacitance ratio”, which can be defined as η:

(11) η=CmaxCmin.

**Figure 3:**
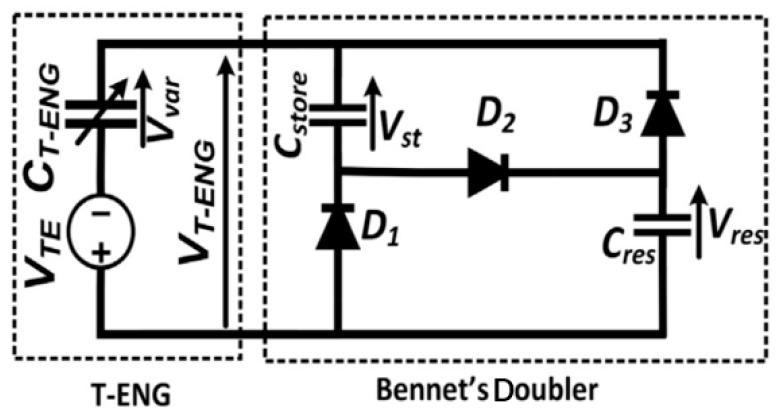
Bennet’s doubler circuit diagram.

The minimum value ($C_{min}$) we measured was 2.8 pF, and the maximum value ($C_{max}$) was 15.8 pF, so η=5.643. In addition, we used Bennet’s doubler circuit to increase the voltage and store the energy, as shown in Figure 3. For η > 2, the Bennet’s doubler works in exponential mode, while for η < 2, the Bennet’s doubler works in saturation mode [34]; thus, in this design, the doubler works in exponential mode.

The Bennet’s doubler circuit, which is a very basic doubler circuit in this area, and it can raise the voltage several times. Through the unidirectional conductivity of the diode, the power stored in the super capacitor is continuously increased, where $V_{TE}$ is the ideal equivalent voltage of Energy Harvester, $C_{T-ENG}$ is the equivalent capacitance, $V_{T-ENG}$ is the equivalent output voltage of Energy Harvester, $C_{store}$ is the energy storage capacitance, $D_{1},D_{2},D_{3}$ are all unidirectional conduction diodes, $C_{res}$ is the filter capacitance.

**Figure 4:**
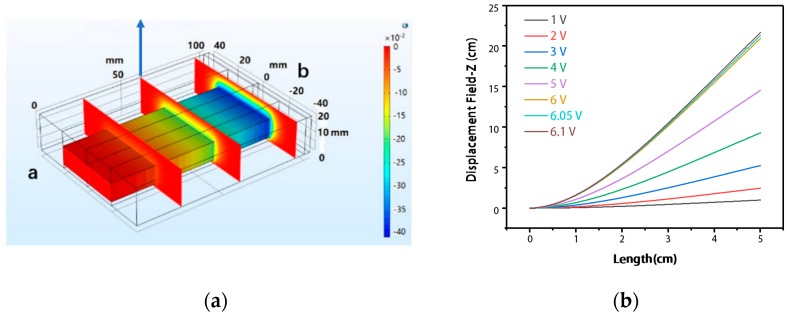
(**a**) Electric field distribution under different displacements. (**b**) Different positions, different displacement corresponding voltages.

We fixed one end of the device, and the other end was used as the active end. By moving the active end and using COMSOL for simulation, the electric field distribution at different positions was obtained in Figure 4a. The voltage in the simulation was generated by moving different displacement at different sampling points of the device. In Figure 4b, the device moves in the direction of the blue arrow on the way. The abscissa of Figure 4b in the article shows the position of the sampling point from the fixed end (b) during the period, and the ordinate shows the displacement of the device along the vertical direction.

**Figure 5:**
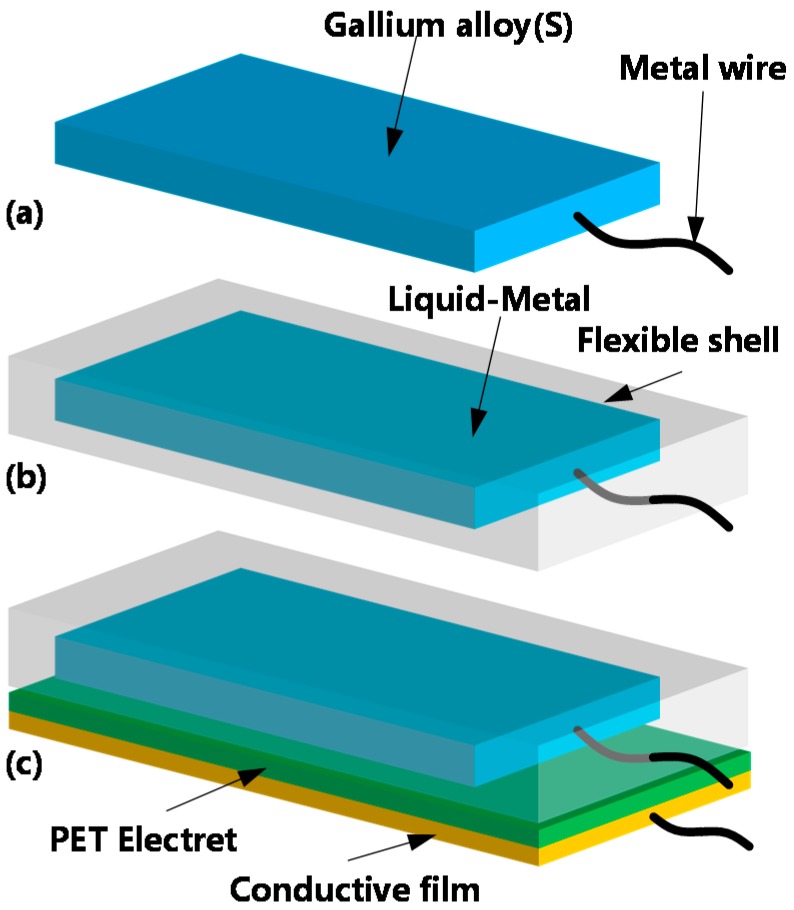
Schematic diagram of the fabrication process of the designed energy harvester. (**a**) molding and low-temperature curing of liquid gallium alloy electrode; (**b**) secondary molding of dragon-skin silicone rubber; (**c**) assembly of the designed energy harvester.

The fabrication process of the designed energy harvester is shown in Figure 5. First, liquid gallium alloy was prepared from a solid-state electrode by the molding method and low-temperature curing. Simultaneously, metal wires were embedded in it, as shown in Figure 5a. Then, using dragon-skin silicone rubber for secondary molding, the solid-state electrode was wrapped in the silicone rubber, as shown in Figure 5b. Then, the electrode was heated to the melting point, and when the metal melted into liquid state, the Ga electrode became stretchable. Finally, the FEP electrode, conductive film and the silicone elastomer made up the whole device, as shown in Figure 5c. With this method, the liquid-metal embedded silicone elastomer could be easily obtained, and the pattern of liquid-metal electrodes could be easily realized.

**Figure 6:**
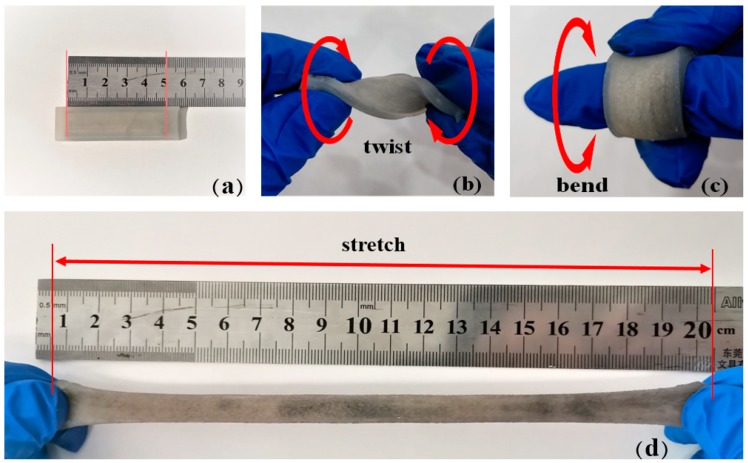
Liquid-metal embedded silicone elastomer has good flexibility and stretchability. (**a**) Fabricated liquid-metal embedded silicone elastomer; Twisting (**b**), bending (**c**), and stretching (**d**) of the fabricated liquid-metal embedded silicone elastomer.

Figure 6 shows that the fabricated liquid-metal embedded silicone elastomer had good flexible and stretchable properties when the temperature was higher than 30 °C than that below 30 °C.

**Figure 7:**
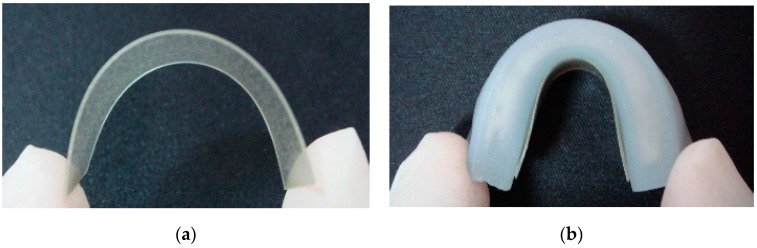
Bonded flexible FEP electret with conductive film (**a**) and fabricated flexible and stretchable energy harvester (**b**).

The bonded FEP electrode and conductive film are shown in Figure 7. The composite film also shows good flexibility to meet the requirements of flexible and stretchable energy harvesters.

## 3. Characterization

The open-circuit voltage (OCV) represents a voltage source’s full voltage. For the designed flexible and stretchable energy harvester, the OCV is mainly affected by three parameters: the liquid alloy content in the electrode, the acceleration generated by external extrusion, and the resistance of the external load. Therefore, in this part, the influence of the above three parameters on the energy output performance of the energy harvester was explored.

### 3.1. Effect of Liquid Composition on Open-Circuit Voltage

**Figure 8:**
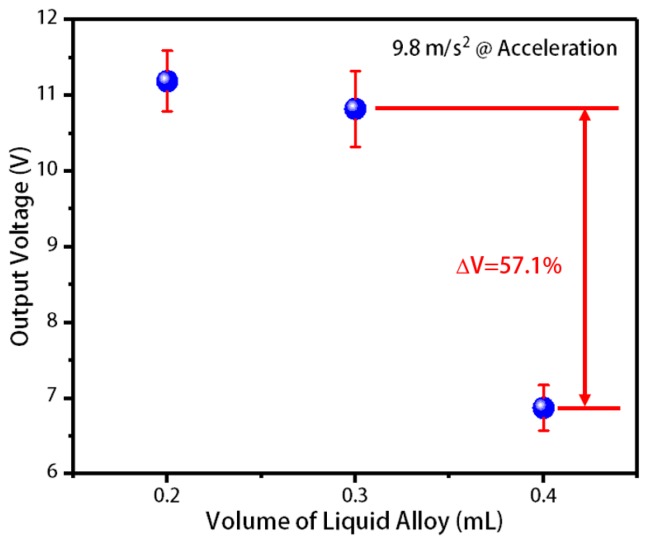
The open-circuit voltage of three groups of flexible and stretchable energy harvesters under the press of gravity acceleration.

The fabricated liquid-metal flexible and stretchable energy harvesters containing 0.2 mL, 0.3 mL, and 0.4 mL liquid alloys were placed on a vibratory table with an external load of 200 MΩ. The press was performed under gravity acceleration, and the open-circuit voltages of the three energy harvesters were measured in Figure 8. As shown in Figure 8, as the liquid alloy content increased, the open-circuit voltage decreased. This is because the capacitance changes of the energy harvester electrode with a lower liquid metal content are larger than the higher one under the same press. This force is the gravity of a 10 g weight that we use. This force and the frequency of vibration are independent of each other. We used the shaking table to generate vibration. We could only set the frequency, and the stroke was different under different frequencies, and the magnitude of the force was also different. The frequency we set was 50–60 Hz. In this case, the vibration frequency and stroke of the shaking table were small, and the power generation efficiency of 0.2 mL liquid metal electrode was higher than that of the other two volumes.

### 3.2. Effect of Extrusion Acceleration on Open-Circuit Voltage

**Figure 9:**
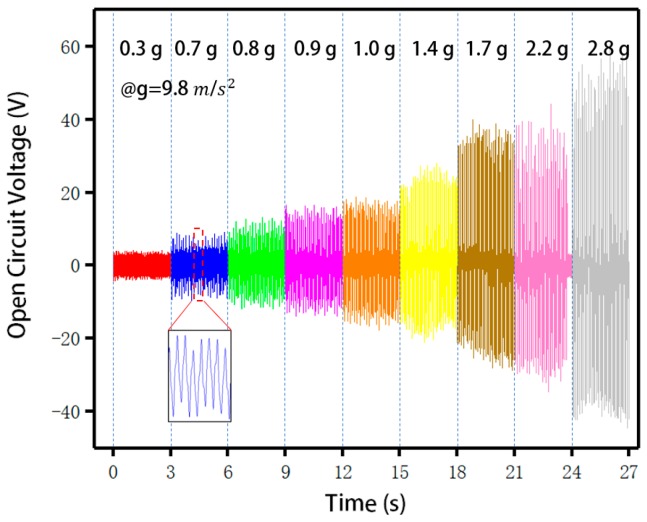
The open-circuit voltage of the flexible and stretchable energy harvester under different press accelerations.

To analyze the influence of extrusion acceleration on the open-circuit voltage, the energy harvester containing 0.4 mL liquid alloy was placed on a vibratory table and externally loaded with a resistance of 200 MΩ. Different open-circuit voltages due to different extrusion accelerations are shown in Figure 9. As shown in the figure, with the increase of the extrusion acceleration, the open-circuit voltage increased significantly; when the acceleration was 0.3 gravity acceleration, the open-circuit voltage was approximately 5 V, and when the acceleration was 2.8 gravity acceleration, the open-circuit voltage increased to 60 V. Since the difference between different devices was only the difference of the volume of the liquid metal alloy, and the resistance of the liquid metal alloy could be ignored compared with the full device. What we used was the matching impedance of the resistance box, which is only a general range, and it cannot be very fine. 200 MΩ was the most impedance we got after testing. The enlarged part of the figure shows the details of the waveform.

### 3.3. Effect of Load Resistance on Open-Circuit Voltage

For the energy harvester, different external loads will result in different output powers, and the output power can only reach its maximum value when the external load and the internal parameters reach the best matching impedance. Therefore, the energy harvester containing 0.2 mL of liquid alloy was tested under the effect of 1.5 accelerations of gravity acceleration to find the optimal matching impedance.

**Figure 10:**
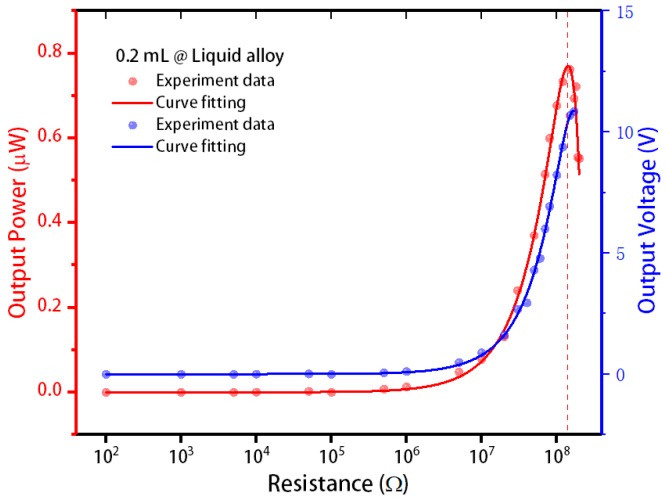
The output performance of stretchable and flexible energy harvesters under different external loads.

The test results are shown in Figure 10. As the external load increased, the output voltage of the energy harvester containing 0.2 mL of liquid alloy gradually increased, and the output power continued to increase slowly. When the load exceeded 10 MΩ, the output voltage and output power increased rapidly, and when the external load reached approximately 200 MΩ, the energy harvester’s output power reached the maximum value of 0.7 μW. Each output corresponded to a different resistance, and we calculated the power according to the following formula: $P=\frac{U^{2}}{R}$.

## 4. Application

The silicone elastomer of the flexible electrode imparts a large tensile property to the fabricated energy harvester, making it extremely adaptable in human body energy harvesting, especially for the energy harvesting of human joint movement.

**Figure 11:**
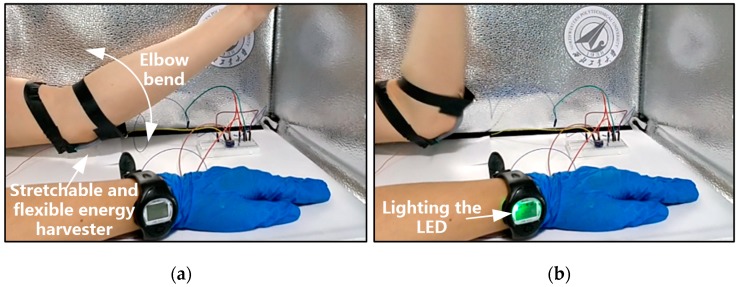
Application of the proposed flexible and stretchable energy harvester in the wearable watch. After wearing the stretchable and flexible energy harvester, the elbow bending action (**a**) could charge the wearable watch (**b**).

In this paper, to verify the effectiveness of the designed stretchable and flexible energy harvester, it was mounted on the elbow of a human body to harvest the energy produced by the bending of the elbow, as shown in Figure 11a. When the elbow was bent, the energy harvester would be squeezed and stretched; according to the working principle shown in Figure 2, the energy harvester converted elbow bending into electrical energy that is stored in a super capacitor. When the capacitor was connected to the watch, the LED in the watch would be lit, as shown in Figure 11b. If we did not use the peripheral circuit to store the generated energy, the LED lit up and went out with the motion. We stored the energy in the super capacitor through Bennet’s doubler circuit, and then used the super capacitor to discharge to achieve the continuous use.

**Figure 12:**
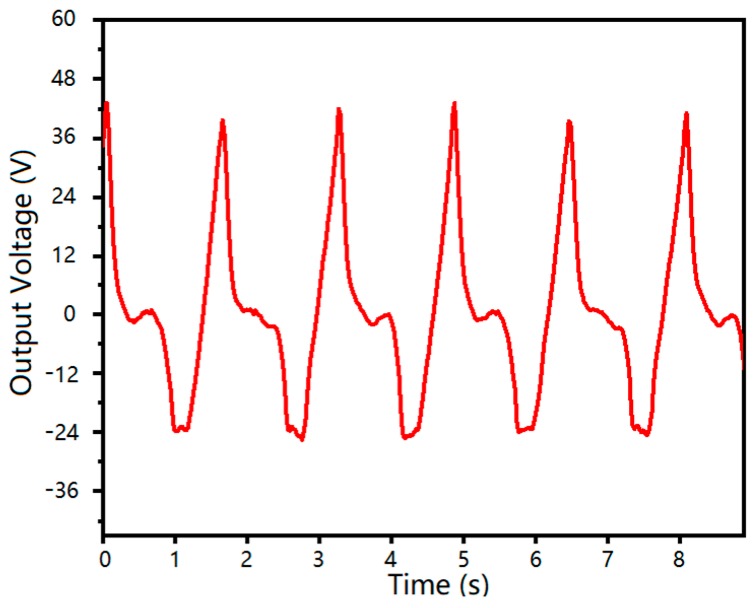
Output voltage of the designed stretchable and flexible energy harvester.

The output voltage and its repeatability of the stretchable and flexible energy harvester are shown in Figure 12. This is a test of the output performance of the generator tied to the arm. The abscissa is the motion frequency of the arm, and the ordinate is the output voltage of the device. It can be seen that the maximum voltage was about 43V and had good repeatability.

**Figure 13:**
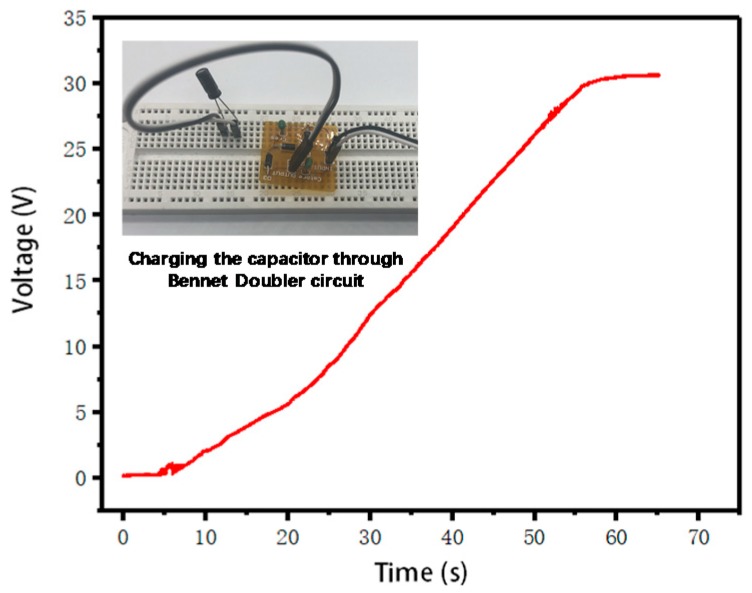
Super capacitor charging curve and Bennet’s doubler circuit (inside).

At present, energy storage is still an important issue. Our group, like others in the field of energy harvesting, used super capacitors for energy storage. We charged the supercapacitor through Bennet’s doubler circuit, and we used the 10 μF capacitor as the energy storage element, which could be filled to 31 V in 50 s, and the charging curve is shown in Figure 13.

## 5. Conclusions

This paper presented the design, fabrication, and application of a novel flexible and stretchable energy harvester based on liquid-metal and FEP electret films. The fabricated liquid-metal stretchable electrode had a stretchability of 400% using dragon-skin silicone rubber. The evaluation parameters of the energy harvester included open circuit voltage and output power. In order to verify the influence of these two parameters on the energy harvester, we made different experiments, and got the following conclusions: in the case of small stroke, the power generation efficiency of 0.2 mL liquid metal alloy was lower than that of 0.4 mL liquid metal alloy. Due to the small stroke and small volume, the distance between the two electrodes changed little. When the formation was large, the power generation effect of 0.2 mL liquid metal alloy electrode was better than that of 0.4 mL liquid metal alloy electrode. The vibratory table test shows that when the acceleration was 2.8 gravity acceleration, the open-circuit voltage could reach 60 V, and the output power was 0.7 μW. The power was still very small and needed further improvement. Experiments show that the proposed flexible and stretchable energy harvester could convert elbow bending into electrical energy and light the LED. As we all know, the wearable technology had evolved to become one of the biggest industries in the world, but frequent charging and battery pollution were key technologies limiting its development. This paper provided a feasible technical solution for these problems. In addition, there were many problems to be solved in the energy storage scheme, such as short charge storage time, large leakage current, and so on. The current solution to these problems in the world was to use the power generated immediately. Recently, a super capacitor with the characteristics of lithium battery was invented, so in the future, we planned to use the lithium ion capacitor produced by the TAIYO YUDEN company as the energy storage device.
